# High-Prevalence Stunting in Preschool Children (1–5 Years) Attending Selected Health Centers in a Food Rich Area-Bushenyi District Southwestern Uganda

**DOI:** 10.1155/2021/5736864

**Published:** 2021-07-20

**Authors:** Douglas Mugarura, Herbert Izo Ninsiima, Hellen Kinyi, Ejike Daniel Eze, Sam Tumwesigire, Prossy Mbekeeka, Andrew Ndamira

**Affiliations:** ^1^Department of Pediatrics and Child Health, Kampala International University Teaching Hospital, Kampala, Uganda; ^2^Department of Physiology, Kabale University School of Medicine, Kabale, Uganda; ^3^Department of Biochemistry, Kabale University School of Medicine, Kabale, Uganda; ^4^Department of Pediatrics and Child Health, Kabale University School of Medicine, Kabale, Uganda

## Abstract

The prevalence of stunting among children in Uganda and Sub-Saharan Africa is still high, and if Uganda is to achieve the food-related Sustainable Development Goals (SDGs), it must urgently invest in improving nutrition and sanitation. In a food rich area like Bushenyi, chronic undernutrition could be due to several other factors than mere scarcity of food. *The Objective(s)*. This study was carried out to determine the prevalence and socioclinical factors responsible for chronic undernutrition (stunting) among preschool children aged 1–5 years in selected Health facilities in Bushenyi district. *Methodology*. This was a cross-sectional study assessing the prevalence of stunting and its associated factors among children aged 1–5 years attending selected health centers in Bushenyi District. Data was collected using a pretested questionnaire, taking anthropometric measurements (height/length), and stool analysis for eggs of soil-transmitted helminthes. Prevalence of stunting was presented as percentages. Logistic regression with adjusted prevalence ratio was performed to test the association between the sociodemographic and clinical factors and stunting at bivariate levels of analysis. *Results*. Most of the children were female, with a median age of 2.1 years and resided in semiurban areas of Bushenyi with their parents. Prevalence of stunting was 89.3%. Only 10.7% of the children were infested with soil-transmitted helminthes. Children likely to be stunted were those who drank unboiled water and were exclusively breastfed. *Conclusion*. There is a high prevalence of chronic malnutrition in Bushenyi district associated with parents'/care takers' low level of knowledge.

## 1. Introduction

Under nutrition is an underlying cause of over half of child deaths. It is associated with lower school enrollment and poor cognitive functioning among children with subsequent effects on performance, and social wellbeing of communities in developing countries like Uganda [1]. Undernutrition indicators include wasting, stunting, and underweight. Stunting or low height-for-age (HAZ) is a good indicator of undernutrition and represents a status of chronic nutritional stress in children [2].

In 2010, it was estimated that 171 million preschool aged children were stunted; 95% of whom lived in developing countries [3]. In Uganda, data from the 2016 Uganda Demographic and Health Survey indicate that 3 in 10 children under the age of 5 are stunted [4]. The proportion of stunted children is highest in Western Uganda with a prevalence of 34.9% [5].

Despite the high levels of malnutrition, 89% of Uganda is defined by the Food and Agricultural Organization (FAO) as being food secure with the Northern and Eastern parts being most vulnerable to food insecurity [6, 7]. However, the prevalence of stunting in children aged under 5 years is lower in the Teso (14.3%) and Lango (22.3%) regions located in Eastern Uganda compared to Bushenyi (29.3%) which is located in the Western region sometimes referred to as “the food basket” of the country [4, 8]. Thus, under nutrition in this region could be due to other underlying problems such as infestation with soil transmitted helminthes (STH), social-economic factors, and lack of nutrition knowledge.

Soil-transmitted helminths (STHs) are intestinal worms transmitted through contaminated foods, water, soil, and hands and thrive in Latin America, tropical and subtropical regions of Asia, and sub-Saharan Africa [9, 10]. Children living in less developed countries like Uganda are likely to be parasitized with at least one STH, resulting in impaired cognitive, intellectual and physical development [2, 11, 12]. Despite Uganda implementing the child day plus campaign, which targets deworming children aged 1–14 years, many children aged 1–5 years are missed out as caretakers fail to attend child day plus [13]. This has resulted in a low treatment coverage and high prevalence of up to 26.5% in Western Uganda in children under the age of 5 [14].

Although the high prevalence of stunting and STH in children under the age of 5 in southwestern Uganda is high, few studies have explored the association of STH and stunting in the region. This study, therefore, aimed at establishing the prevalence of stunting and its association with STH and other socioclinical factors among children (1–5 years) attending selected health units in Bushenyi district of southwestern Uganda.

## 2. Methods

### 2.1. Study Area

Bushenyi district is located in the western region of Uganda, about 323 km from the capital city Kampala. The district is made up of 11 subcounties, 76 Parishes, and a total of 585 villages with an estimated total population of 235,617 people as per the National population and housing census of 2016. The district can be divided into two regions; semiurban and rural. The semiurban region is located within the Bushenyi Ishaka Municipality and is the administrative part of the district. The rest of Bushenyi is rural, with agriculture being the commonest economic activity employing 86.7% of the population [4]. The four health units selected for the study serve the rural and semiurban populations of the greater Bushenyi district.

### 2.2. Study Design

This was a cross-sectional study carried out on preschool children attending the outpatient departments of 4 health units located in Bushenyi district. They included Kampala International University Teaching Hospital (Teaching hospital of Kampala International University) Bushenyi Health center IV, Kyabugimbi Health center IV, and Kyamuhunga Health Center IV. A pretested questionnaire was used to collect data from the caretakers of the children who fit the inclusion criteria. The children's weight and height were then taken, and with the help of the caretaker, a stool sample was collected from the child.

### 2.3. Study Population and Sampling

The study population comprised all the preschool children attending the four health centers. The study participants were 206 preschool children aged 1–5 years old who attended the selected health centers in Bushenyi District from the 20th March to 30th of July 2019.

Sample size was determined using the single population formula. It was calculated by using a previous prevalence of 17.4% (Zemene et al. 2018) with a margin of error of 0.05 and a confidence level of 95%. In line with it, 220 children was the minimum sample size. Children attending the health units, on dates of data collection phase of the study, were consecutively recruited into the study. However, only 206 children fit the inclusion criteria.

Respondents were caretakers of children aged between 1 and 5 years that had brought them to the health unit. Children excluded from the study were those who were too sick, unable to give stool samples, whose caretaker refused to give ethical consent, and those who had been dewormed 2-3 months prior to the date of the study.

### 2.4. Socioclinical Data Collection

An interview based structured questionnaire was used to collect socioclinical data. The questionnaire was initially developed in English and translated to Runyankole, the local language and then retranslated back to English for analysis to ensure the consistency. A pretest was administered on caretakers of 10 preschool aged children attending the pediatric clinic of Kampala International University, Teaching Hospital, and relevant amendments were made. Written informed consent was obtained from each caregiver before the interview was conducted.

### 2.5. Assessment for Stunting

The height and weight of each child that fulfilled the inclusion criteria were taken, recorded using a stadiometer (SECA®) and infantometer (SECA®), and interpreted using WHO Z-scores [15]. For children aged above two years, the child stood on a precalibrated weighing scale with his/her back against the weighing scale board, his/her heels, buttocks, shoulders, and head touching a flat upright head piece. The child was instructed to place their feet, knees, and ankles together. The head piece was brought down onto the upper most point on the head, and the height was read to the nearest 0.1 cm at the examiner's eye level. For those children below 2 years, length was taken using an infantometer. The child would lie on it, and a length read and recorded to the nearest 0.1 cm [16]. The weight of the children was read to the nearest 0.1 g.

### 2.6. Stool Sample Collection and Processing

The caregiver was provided with gloves and a stool container labeled with the child's study number. He/she was instructed on how to collect a stool sample using the applicator provided in a specimen container. The child and caregiver used a side room near the laboratory to collect the stool sample, after which soap and clean water were provided to their wash hands.

In the laboratory, one gram of stool sample was placed in a screw capped bottle and emulsified in 8 mls of 10% formaldehyde in water. The emulsion was sieved, and the suspension transferred into a glass tube, where 3 mls of diethyl ether was added. These were then mixed and centrifuged at 750 revolutions for a minute. Using a stick, any fecal debris layer was made loose and decanted to remain with the sediment at the bottom. The sediment was transferred onto a microscopic slide, and a cover slip applied. It was then mounted to a light microscope using the 10x objective and 40x objective to examine for soil-transmitted helminthes' eggs [17]. When found, the eggs were confirmed using an Atlas and further by a laboratory technologist.

### 2.7. Statistical Analysis

Data from precoded and completed questionnaires was entered into Microsoft excel, cleaned, and analyzed using Statistical Analysis Software (STATA 12.0). The prevalence of children with stunting was calculated and presented as percentages. Information on sociodemographic and clinical factors was descriptively presented as frequencies (percentages). A logistic regression with adjusted Prevalence Ratio was performed to test the association between the sociodemographic and clinical factors and stunting at a bivariate level of analysis. 95% Confidence Interval was used to assess the strength of association. A *P* value of ≤0.05 was accepted as statistically significant.

### 2.8. Ethical Considerations

Ethical approval was obtained from the Institutional Research Ethics Committees (IREC-Mbarara University Science and Technology (approval number 20/10-16). At all respective health units, a verbal approval by the health unit's leaders was obtained on presentation of an IREC approval document. A written informed consent was obtained from each caregiver/child before the interview was conducted. The questionnaires were administered after attending to the primary reasons in order not to interfere with the patients care. The principal investigator participated in the children's general medical care at outpatient department at the time of data collection. The interviews were carried out in a side room to ensure confidentiality. Names were not written on the questionnaires, and only research numbers were used. No individual-based data was reported. After the interview, the researcher replied to any concerns the caregiver had. The completed questionnaires were kept under lock and key and only accessed by the principal investigator. Those children found with soil-transmitted helminthiasis were treated, and those that were stunted a nutritional education were given to their caregivers.

## 3. Results

### 3.1. Prevalence of Stunting

The proportion of children with stunting was 89.3% (95%CI 83.3–92.9).

### 3.2. Demographic Characteristics of the Children Enrolled in the Study

Children attending the selected health centers in the period of 25th to 30th of July 2019 were mainly female (59.7%) aged above two years, with a median age of 2.1 years (IQR = 1.6–2.8). Most children (92.2%) stayed with their parents and resided within semiurban area of Bushenyi (54.9%). Many of the children lived within an extended family (52.4%), and 92.2% had been brought to the health center by their parents who were married (71.8%) as summarized in Table 1.

### 3.3. Prevalence of Clinical Factors Associated with Malnutrition

Although most of the children were dewormed twice a year (64.1%), they did not wash their hands after using the toilet and before eating (84.6%). Furthermore, a majority of them (76.2%) rarely wore shoes. Almost all of the caregivers (96.1%) reported that they had never received any form of education on how to feed their children. However, many of the children (74.3%) had or were being exclusively breastfed, and half of them (50.5%) drank boiled water. A few of the children (10.7%) were infested with soil-transmitted helminthes. These factors are summarized in Table 2.

### 3.4. Bivariate Analysis of the Socioclinical Factors

Children aged above two years and who lived in an extended family setting were less likely to be stunted. On the other hand, children likely to be stunted were those who drank unboiled water (aPR = 1.21, 95%CI 1.10–1.34) and were exclusively breastfed (aPR = 1.35, 95% CI 1.11–1.65) as shown in Table 3.

## 4. Discussion

The 89.3% prevalence of stunting found in this study is high compared to the national and regional prevalence of 29% and 34.9%, respectively [4, 5]. The study prevalence of stunting is also higher than that of a community study conducted in Bushenyi that showed 46% stunting [16]. While the study of [16] focused on households in Bushenyi and assessed socioeconomic and public health factors contributing to chronic malnutrition, our study assessed the contribution of age and sex of child, infestation with STH, and nutrition education of the caregiver to stunting. In addition, our study focused on children attending health centers who are likely to have comorbid conditions that may hinder their growth.

STH remain major public health problems of children in developing countries [2]. The prevalence of 10.7% of STH found in children enrolled in this study is comparable to the national prevalence of 8.8% [18]. However, this study shows that infestation with STH does not increase the chances of developing chronic undernutrition. This finding agrees with that of [19]. In contrast, some studies on preschool aged children found that nearly all infested children had significant stunted growth [2, 20]. This study showed that children who lived in an extended family setting were less likely to be stunted. This could be explained by the attention given to the child by different household members. However, Kikafunda et al. reported that children staying with a large number of family members were likely to be stunted probably due to the competition for resources.

Poor personal hygiene practices such as eating with unwashed hands, consumption of unclean fresh foods, walking barefoot, and consumption of unboiled water may contribute to stunting [3]. This study found that consumption of unboiled water is associated with stunting. Unboiled water could be a vehicle for transmitting both helminthes and other pathologic organisms that impair absorption and utilization of nutrients [12].

This study shows that exclusive breastfeeding had significant influence on stunting compared with the other factors. However, a cross-sectional study done among Malawian infants (below 6 months) showed that exclusive breastfeeding had prevented stunting [21]. This may be due to different age groups as most of the children in this study were aged above 2 years. Children aged above six months need to be supplemented with solid food, which is adequate for their proper growth and development. Finally, our results suggest that the risk of stunting continues to reduce as age increases. This may be due related to feeding habits in this age group. The limitation of this study is that it was based at health centers and assessed children who may have had other comorbidities that may have contributed to stunting.

## 5. Conclusion

The prevalence of stunting in preschool children aged 1–5 years attending health centers in Bushenyi district is extremely high. In this population of children, stunting is likely to occur if the child is less than two years, stays in a nuclear family setting, and drinks unboiled water.

## Figures and Tables

**Table 1:** Sociodemographic and clinical factors of children aged 1–5 years attending the selected health units in Bushenyi district.

Characteristic	Summary measure	Confidence intervals 95%
*Age of children*		
Less than 2 years	86 (41.7%)	21.5–78.7
More than 2 years	120 (58.3%)	51.4–64.9

*Residence*		
Semiurban	113 (54.9)	48.0–61.6
Rural	93 (45.1)	38.4–52.0

*Gender*		
Male	83 (40.3)	33.8–47.1
Female	123 (59.7)	52.9–66.3

*Family*		
Extended	108 (52.4)	45.6–59.2
Nuclear	98 (47.60)	40.8–54.4

*School going*		
No	162 (78.6)	72.7–83.8
Yes	44 (21.40)	16.2–27.4

*Caregiver*		
Parent	190 (92.2)	88.0–95.3
Guardian	16 (7.80)	46.7–12.0

*Marital status of caregiver*		
Single	55 (26.70)	21.7–32.2
Married	148 (71.80)	66.2–77.0
Others	3 (1.50)	0.40–37.2

**Table 2:** Clinical factors associated with malnutrition in children aged 1–5 years attending the selected health units in Bushenyi district.

Characteristic	Summary measure	95% CI
*Nutritional education of caregiver*	Number (%)	
No	198 (96.1)	92.8–98.2
Yes	8 (3.9)	1.8–7.2

*Deworming of child*		
Once a year	74 (35.90)	29.6–42.7
Twice a year	132 (64.10)	57.4–70.4

*Washing hands after toilet and before eating*		
No	28 (13.4)	9.4–18.8
Yes	178 (84.6)	81.2–90.1

*Wearing shoes always*		
No	157 (76.2)	70.0–81.7
Yes	49 (23.8)	18.2–30.0

*Presence of helminths eggs in stool*		
Yes	22 (10.7%)	7.0–15.5
No	184 (89.3%)	84.5–93.0

*Drinking boiled water*		
Yes	104 (50.5%)	43.7–57.3
No	102 (49.5%)	42.7–56.3

*Exclusive breastfeeding*		
Yes	153 (74.3%)	67.4–80.1
No	44 (21.4%)	16.2–27.4
Do not know	9 (4.3%)	2.2–7.9

**Table 3:** Bivariate analysis of major sociodemographic and clinical factors associated with stunting among children attending selected health units in Bushenyi district.

Parameter	Variable	Frequency of stunting			*P* value	Adjusted ratio	Prevalence	Confidence interval 95%
		Absent	Present	Total		Value		95% CI
Sex	Male	10	73	83	0.61	1.00	0.93–1.13	0.88–1.08
	Female	12	111	123		1.03		

Age	≤2	4	82	86	**0.011**	1.00	0.82–0.97	1.02–1.24
	>2	18	102	120		**0.89**		

Family setting	Nuclear	7	101	108	**0.047**	1.00	**0.82**–**1.00**	1.00–1.22
	Extended	15	83	95		**0.91**		

Water drank	Boiled	20	84	104	**<0.001**	1.00	**1.10**–**1.34**	0.75–0.91
	Not boiled	2	100	102		**1.21**		

Exclusive breastfeed	No	13	31	44	**0.002**	1.00	**1.11**–**1.65**	0.61–0.90
	Yes	7	146	153		**1.35**		

Wear shoes always	No	9	40	49	0.11	1.00	0.98–1.29	0.75–1.00
	Yes	13	144	157		1.12		

Helminths egg in stool	No	21	163	184	0.16	1.00	0.97–1.20	0.84–1.03
	Yes	1	21	22		1.08		

The bold values represent those with significant *P* values.

## Data Availability

All the data are included in the manuscript.

## Additional Points

We recommend continuous community health education on proper feeding of children and sanitation. Further research involving the whole community should be done to get more accurate prevalence and intensity of soil-transmitted helminthes.
